# Analysis of Molecular Imaging and Laboratory Baseline Biomarkers in PSMA-RLT: Whole-Body Total Lesion PSMA (TLP) Predicts Overall Survival

**DOI:** 10.3390/cancers16152670

**Published:** 2024-07-26

**Authors:** Connor Hein, Caroline Burgard, Arne Blickle, Moritz B. Bastian, Stephan Maus, Andrea Schaefer-Schuler, Manuela A. Hoffmann, Mathias Schreckenberger, Samer Ezziddin, Florian Rosar

**Affiliations:** 1 Department of Nuclear Medicine, Saarland University—Medical Center, 66421 Homburg, Germany; s8crhein@stud.uni-saarland.de (C.H.); caroline.burgard@uks.eu (C.B.); arne.blickle@uni-saarland.de (A.B.); moritz.bastian@uks.eu (M.B.B.); stephan.maus@uks.eu (S.M.); andrea.schaefer@uks.eu (A.S.-S.); samer.ezziddin@uks.eu (S.E.); 2 Department of Nuclear Medicine, Johannes Gutenberg University, 55101 Mainz, Germany; manhoffm@uni-mainz.de (M.A.H.); mathias.schreckenberger@unimedizin-mainz.de (M.S.)

**Keywords:** biomarker, total lesion PSMA (TLP), prostate-specific membrane antigen (PSMA), PET/CT, radioligand therapy (RLT), metastatic castration-resistant prostate cancer (mCRPC)

## Abstract

**Simple Summary:**

Prostate specific membrane antigen (PSMA)-targeted radioligand therapy (RLT) is a promising and recently approved treatment option for patients with metastatic castration-resistant prostate cancer (mCRPC). The aim of this study was to analyze which laboratory and PET imaging parameters are able to predict biochemical response and overall survival with this treatment. Two quantitative imaging biomarkers were identified that allow prediction of RLT outcome, further improving the pre-therapeutic characterization of mCRPC patients undergoing PSMA-RLT.

**Abstract:**

The aim of this retrospective study was to identify pre-therapeutic predictive laboratory and molecular imaging biomarkers for response and overall survival (OS) in patients with metastatic castration-resistant prostate cancer (mCRPC) treated with prostate-specific membrane antigen (PSMA)-targeted radioligand therapy (RLT). Pre-therapeutic laboratory and [^68^Ga]Ga-PSMA-11 PET/CT data of *n* = 102 mCRPC patients receiving [^177^Lu]Lu-PSMA-617 RLT within a prospective registry (REALITY Study, NCT04833517) were analyzed including laboratory parameters such as alkaline phosphatase (ALP), prostate-specific antigen (PSA), gamma glutamyl transferase (GGT), glutamate oxaloacetate transaminase (GOT), glutamate pyruvate transaminase (GPT), neuron specific enolase (NSE), hemoglobin (Hb), and imaging parameters such as maximum standardized uptake value of the tumor lesions (SUV_max_), the mean standardized uptake value of all tumor lesions (SUV_mean_), the whole-body molecular tumor volume (MTV), and the whole-body total lesion PSMA (TLP). Mann–Whitney U test, univariate and multivariable Cox-regression were performed to test for association of the parameters with response and OS. The SUV_mean_ of all lesions was significantly different between responders and non-responders (SUV_mean_ responders 8.95 ± 2.83 vs. non-responders 7.88 ± 4.46, *p* = 0.003), whereas all other tested biochemical and imaging parameters did not reveal significant differences. Hb and the molecular imaging parameters MTV and TLP showed a significant association with OS (*p* = 0.013, *p* = 0.005; *p* = 0.009) in univariant Cox regression; however, only TLP remained significant in multivariable analysis (Hazard ratio 1.033, *p* = 0.009). This study demonstrates a statistically significant association between the quantitative PET/CT imaging parameter SUV_mean_ and PSA response, as well as between the baseline TLP and OS of mCRPC patients undergoing RLT.

## 1. Introduction

Prostate cancer (PC) is one of the most common cancers worldwide [1]. While a localized carcinoma is associated with a good prognosis, life expectancy is severely reduced if metastasis and castration resistance occur [2,3,4]. Besides androgen receptor signaling inhibitors (ARSI) such as abiraterone and enzalutamide [5,6], and chemotherapy with docetaxel [7] or cabazitaxel as second-line treatment [8], radioligand therapy (RLT) is another established therapeutical option for metastatic castration-resistant prostate cancer (mCRPC). The prostate-specific membrane antigen (PSMA), overexpressed by prostate cancer cells [9,10,11], is the central target structure for molecular imaging and RLT of prostate cancer [12]. Many retrospective and prospective studies have demonstrated the efficacy of PSMA-RLT with positive effect on overall survival (OS) [13,14,15]. Recently, PSMA-targeted RLT with the beta-emitting ^177^Lu was approved by the EMA and FDA [16,17]. RLT is preferably used in cases of high PSMA expression [18] and consequently a PSMA-targeted positron emission tomography (PET/CT) scan, e.g., [^68^Ga]Ga-PSMA-11 PET/CT, is mandatory prior to initiating RLT to assess PSMA expression [19]. The evaluation of prognostic pre-therapeutic tests is a current field of research, as it has the potential to improve patient assessment and represents an important step towards future personalized cancer medicine. The aim of this study was to identify pre-therapeutic predictive molecular imaging and laboratory biomarkers for response and OS in patients with mCRPC treated with [^177^Lu]Lu-PSMA-617 RLT.

## 2. Materials and Methods

### 2.1. Patient Cohort

In this single center retrospective analysis, a total of 102 patients with mCRPC receiving [^177^Lu]Lu-PSMA-617 RLT at our institution were investigated regarding pre-therapeutic predictive molecular imaging and laboratory biomarkers. Comprehensive laboratory results and [^68^Ga]Ga-PSMA-11 PET/CT scans, ensuring adeqaute PSMA expression, had to be available prior to RLT. All patients participated in the “prospective registry to assess outcome and toxicity of targeted radio-nuclide therapy in patients with mCRPC in clinical routine (REALITY Study)”, NCT04833517 and the analyzed cohort was congruent with that of a previously published study by our working group [20]. The study was approved by the Institutional Review Board of Ärztekammer des Saarlandes/Saarbrücken (ethics committee permission number 140/17). Written informed consent was obtained from all study participants. Patients received multiple prior treatments such as ARSI or chemotherapy. Detailed patient characteristics are summarized in Table 1.

### 2.2. Therapy Details and Response Assessment

Each patient received at least two cycles of [^177^Lu]Lu-PSMA-617 RLT at our institution. Administered [^177^Lu]Lu-PSMA-617 was synthesized according to standard procedures described by Kratochwil et al. [21]. ^177^Lu and PSMA-617 was obtained from IDB Holland BV (Baarle-Nassau, The Netherlands), and ABX advanced biochemical compounds GmbH (Radeberg, Germany), respectively. The median number of RLT cycles was 5 (range 2–18). In total a median cumulative activity of 32.0 GBq ^177^Lu (range 7.6–109.2 GBq) was administered per patient. Injected activities were individually adjusted to the patient’s characteristics, such as distribution and extent of tumor burden, tumor progression, body surface area, and renal function. Median administered ^177^Lu activity per RLT cycle was 7.0 GBq and in the range 1.1–11.6 GBq. Prostate-specific antigen (PSA) values were closely monitored during and after treatment. Patients who responded to treatment were defined as patients who achieved partial remission with a PSA drop of ≥50%, measured as the maximum decrease at any time during the course of PSMA-RLT.

### 2.3. Serum Biomarker

Blood samples were collected right before the start of RLT. Analyzed serum biomarkers included alkaline phosphatase (ALP), prostate-specific antigen (PSA), gamma glutamyl transferase (GGT), glutamate oxaloacetate transaminase (GOT), glutamate pyruvate transaminase (GPT), neuron-specific enolase (NSE), and hemoglobin (Hb).

### 2.4. PET/CT Imaging and Imaging Biomarkers

Baseline [^68^Ga]Ga-PSMA-11 PET/CT was performed 14 ± 13 days before the start of the first [^177^Lu]Lu-PSMA-617 RLT cycle with a mean activity of 132.5 MBq (range 77–195 MBq). The PSMA ligand PSMA-11 was purchased from ABX advanced biochemical compounds GmbH (Radeberg, Germany) and ^68^Ga from Eckert & Ziegler Strahlen- und Medizintechnik AG (Berlin, Germany) using a ^68^Ga/^68^Ge generator. Following the guidelines for prostate cancer imaging [22], the time between injection of the tracer and imaging was 60 min. All PET/CT scans were performed using a Biograph 40 mCT PET/CT scanner (Siemens Medical Solutions, Knoxville, TN, USA). The acquisition time was 3 min/bed position, the slice thickness was 3.00 mm, and an expanded field of view of 21.4 cm (TrueV) was used. For attenuation correction and anatomical localization, a low-dose CT was acquired. A three-dimensional OSEM algorithm with 3 iterations, 24 subsets, Gaussian filtering, and a slice thickness of 5.00 mm was used for PET reconstruction. 

**Figure 1:**
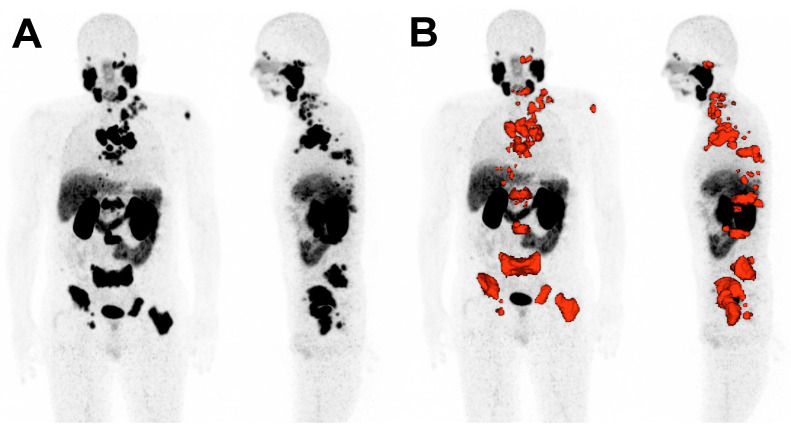
Representative example of tumor delineation using Syngo.Via software (VB 40B). (**A**): Maximum intensity projection (MIP) of [^68^Ga]Ga-PSMA-11 PET/CT. (**B**): Tumor tissue is delineated in red.

The imaging biomarkers to be analyzed included the maximum standardized uptake value of the tumor lesions (SUV_max_), the mean standardized uptake value of all tumor lesions (SUV_mean_), the whole-body molecular tumor volume (MTV), and the whole-body total lesion PSMA (TLP), which is the analogue parameter to the established total lesion glycolysis (TLG) on [^18^F]FDG PET/CT [23,24]. TLP was defined as the summed products of volume and uptake (∑ Volume × SUV_mean_) of all lesions. MTV and TLP were calculated by a semi-automated tumor segmentation algorithm using Syngo.Via software (Enterprise VB 40B, Siemens, Erlangen, Germany). A standardized uptake of SUV ≥ 3 was used as a threshold for delineation of tumor lesions as described by Ferdinandus et al. [25]. Lesions that fell below a volume < 0.2 mL were excluded. Physiological uptake, e.g., in the bladder, spleen, liver or salivary glands, was manually excluded. Liver metastases were segmented by a threshold value of 1.5 × SUV_mean_ of non-metastatic liver tissue. Figure 1 depicts exemplarily tumor segmentation using Syngo.Via software (VB 40B). 

### 2.5. Statistical Analysis

For statistical analysis, SPSS Version 29.0.2 (IBM Corp., Armonk, NY, USA) and Prism Version 8.2.0 (GraphPad Software, San Diego, CA, USA) were used. Besides descriptive statistics, Mann–Whitney U Test, survival analysis, univariate and multivariable Cox regression were performed to test for association with response and OS. OS was defined as the time between the start of PSMA-RLT and either death or the last study visit. Patients were contacted at regular intervals with a cutoff date of 2 May 2023. A *p*-value < 0.05 was regarded as statistically significant. Variables that contributed to the univariate Cox regression model (*p* < 0.05) were included in the multivariable Cox regression analysis, using a stepwise backward elimination model to identify independent baseline predictors of OS.

## 3. Results

### 3.1. Baseline Laboratory and Biochemical Parameters and Predictors for PSA Response

The biochemical parameters, determined by laboratory testing (ALP, Hb, PSA, NSE, GGT, GOT, and GPT) and imaging parameters determined by [^68^Ga]Ga-PSMA-11 PET/CT (SUV_max_, SUV_mean_, MTV, and TLP) at baseline, i.e., before start of [^177^Lu]Lu-PSMA-617 RLT are presented in Table 2.

In the study cohort, 70/102 patients (68.6%) showed a response during course of RLT with a PSA-decrease ≥ 50%, while 32 patients (32.4%) showed no response and were therefore categorized as non-responders. Of all tested baseline laboratory and molecular imaging parameters, only the molecular imaging parameter SUV_mean_ revealed a significant difference between responders and non-responders (8.95 ± 2.83 vs. 7.88 ± 4.46, *p*-value = 0.003). Table 2 summarizes the results for all tested parameters. 

### 3.2. Predictors for Overall Survival

Median OS including the entire patient cohort was 16.8 months (95% CI 13.6–19.9 months). The median follow-up time was 44.4 months (95% CI 23.5–65.3 months). For laboratory parameters, univariate Cox regression revealed only a significant association with OS for Hb (HR 0.852, 95% CI 0.75–0.967; *p* = 0.013). Other laboratory parameters did not demonstrate a significant association with OS (all *p*-values ≥ 0.158). Similarly, the imaging parameters SUV_mean_ and SUV_max_ did not reveal a significant association with OS. In contrast, whole body molecular imaging parameters, i.e., MTV (HR 1.324, 95% CI 1.088–1.611; *p* = 0.005) as well as TLP (HR 1.033, 95% CI 1.008–1.059; *p* = 0.009) were significantly associated with OS. 

**Figure 2:**
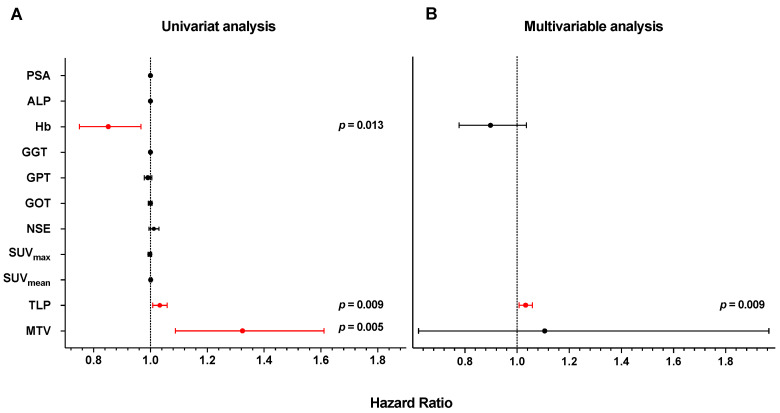
Hazard ratios for OS of tested parameters for (**A**) univariate and (**B**) multivariable Cox regression.

The variables contributing to the univariate analysis were included in a multivariable Cox regression. In multivariable analysis, only TLP remained a significant independent variable associated with OS (HR 1.033, 95% CI 1.008–1.059, *p* = 0.009), whereas Hb and MTV could not be outlined as independent variables associated with OS. Figure 2 and Table 3 summarize the results of the univariate and multivariable analyses. 

**Figure 3:**
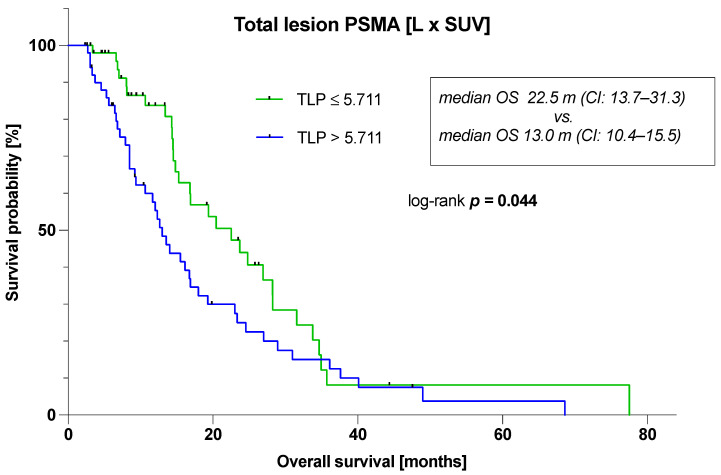
Kaplan–Meier-plot illustrating overall survival stratified by median value of total lesion PSMA (TLP).

Figure 3 depicts a Kaplan–Meier curve for the TLP, which has been tested significant in the multivariable analysis, stratified by the corresponding median value. Patients with a TLP > 5.711 L × SUV experienced a significantly shorter OS (log-rank *p* = 0.044) with median 13.0 (95% CI: 10.4–15.5) vs. 22.5 months (95% CI: 13.7–31.3), respectively. In addition, Kaplan–Meier log-rank analysis revealed that OS was significantly longer (*p* < 0.001) in PSA responders (median 23.3 months; 95% CI: 15.4–31.3) compared to PSA non-responders (median 8.1 months; 95% CI: 3.9–12.3).

## 4. Discussion

The aim of this study was to identify predictive pre-therapeutic biochemical and molecular imaging factors for response to [^177^Lu]Lu-PSMA-617 RLT and OS. In a cohort of *n* = 102 patients participating in the REALITY study (NCT 04833517), our results reveal mean lesional tracer uptake on PSMA PET/CT as significant prognostic parameter for response to PSMA-RLT, whereas PSMA PET/CT-based total tumor burden showed to be an independent predicator for OS.

Considering a PSA response of ≥50%, the only predictive biomarker found was the mean standardized uptake (SUV_mean_) of ^68^Ga-PSMA-11 of all lesions on PET/CT (*p* = 0.003), whereas all other tested biochemical and imaging parameters showed no predictive value for response. This is in line with a phase 2 pilot study by Emmett et al. [26] and other retrospective studies in mCRPC patients, which were able to predict a PSA reduction utilizing SUV_mean_ [27,28,29]. The increased mean lesional uptake in patients responding to therapy may suggest an increased accumulation of the therapeutic agent [^177^Lu]Lu-PSMA-617, which possibly explains the higher response rates during the course of RLT. Widjaja et al. reported the mean lesion SUV_max_ to be a predictive factor for early PSA response after two cycles of RLT [18]. In contrast, our study using SUV_max_ of the lesion with the highest tracer uptake does not show such predictive value of this imaging biomarker. Predicting PSA response does not appear to be possible using the TLP (*p* = 0.199), suggesting that the level of tumor burden at baseline is not associated with therapy response. Alternatively, Zou et al. demonstrated that a determination of the imaging biomarker TLP enables independent prediction of PFS based on PSA value [30].

Besides response prediction, the prediction of OS is essential. Various studies have investigated baseline laboratory values to identify potential pre-therapeutic biomarkers. For example, ALP or GOT values have proven to be suitable for predicting OS [25,31,32]. Interestingly, in the present study we found no significant association between OS and most of the analyzed pre-therapeutic laboratory values, with the exception of Hb, which showed an impact on OS (*p* = 0.013). This impact of Hb on OS is in line with a report by Dai et al. [33]. The molecular imaging parameters MTV and TLP both showed a significant association with OS (*p* = 0.005; *p* = 0.009), with only TLP remaining in multivariable Cox regression analysis. Further image-derived parameters, such as SUV_max_ and SUV_mean_, did not show to be a predictive parameter in our study, as the most uptake-intensive metastasis is not a suitable predictor of OS; a similar finding has been reported by Seifert et al. [34]. Thus, our results suggest that for OS prediction, the imaging biomarker TLP seems to be superior to all other tested biochemical and imaging biomarkers. We anticipate the predictive value of TLP is based on its ability to assess molecular imaging characteristics as PSMA expression and volumetric burden of prostate cancer, as it is calculated by the summed products of volume × uptake (SUV_mean_) of all lesions. 

Concluding, quantitative analysis of imaging parameters could have several advantages, such as the prediction of PSA response based on the SUV_mean_ value, or the prognosis of the OS based on the TLP value. Considering these findings, we suggest that the parameter SUV_mean_ as well as TLP determination should be established in clinical practice and evaluated at baseline of PSMA-RLT. In terms of implementing the TLP determination, difficulties could arise due to the time-intensive segmentation process with up to 20–30 min per patient. However, artificial intelligence-supported algorithms could potentially perform TLP segmentation in a more efficient manner in the future, which would enable convenient implementation in daily clinical practice [35].

Despite the promising results, there are certain limitations to be noted. The retrospective study design, monocentric analysis, and the limited number of patients may impact and decrease generalizability of the results. Especially, the limited number of patients precluded more detailed subgroup analyses, which should be addressed in subsequent studies. Future studies should be conducted in a prospective setting and with a larger patient cohort. Furthermore, in this study, TLP segmentation was performed according to the method proposed by Ferdinandus et al. with a fixed SUV threshold of ≥3 for delineation of tumor lesions [25]. Besides this method, TLP segmentation can be determined using other approaches, such as the determination with relative and non-fixed threshold values; e.g., 41% or 50% of SUV, as used in FDG PET imaging [36], which may lead to different results. 

## 5. Conclusions

This study demonstrates a statistically significant association between the quantitative PET/CT imaging parameter SUV_mean_ and PSA response, as well as between the baseline TLP and OS of mCRPC patients undergoing RLT. Quantitative image analysis of whole-body tumor burden appears valuable for pre-therapeutic characterization and promises translation into clinical practice.

## Figures and Tables

**Table 1:** Patient characteristics.

Age	Median [years] (Range)	72 (48–88)
PSA	Median [ng/mL] (range)	130 (2–9579)
ALP	Median [U/L] (range)	109 (22–1753)
ECOG performance status	*n* (%)	
0		29 (28.4)
1		51 (50.0)
≥2		22 (21.6)
Sites of metastases	*n* (%)	
Bone		93 (91.2)
Lymph node		79 (77.5)
Liver		17 (16.7)
Other		29 (28.4)
Prior therapies	*n* (%)	
Prostatectomy		51 (50.0)
Radiation		63 (61.8)
ADT		102 (100)
ARSI		97 (95.1)
Abiraterone		74 (72.6)
Enzalutamide		84 (82.4)
Abiraterone and Enzalutamide		61 (59.8)
Chemotherapy		67 (65.7)
Docetaxel		66 (64.7)
2nd line cabazitaxel		28 (27.5)
[223Ra]Ra-dichloride		18 (17.7)
PSMA-RLT cycles	Median (range)	5 (2–18)
Cumulative activity of ^177^Lu	Median [GBq] (range)	32.0 (7.6–109.2)

androgen deprivation therapy (ADT); alkaline phosphatase (ALP), androgen receptor signaling inhibitors (ARSI); Eastern Cooperative Oncology Group (ECOG); prostate-specific antigen (PSA).

**Table 2:** Pre-therapeutic laboratory and molecular imaging parameters tested for predication of PSA response.

Variable	All Patients		Responders		Non-Responders		*p*-Value
	Mean ± SD	Range	Mean ± SD	95% CI	Mean ± SD	95% CI	
**Serum**							
ALP [U/L]	185 ± 244	22–1753	198 ± 288	130–267	155 ± 87	124–186	0.166
Hb [g/dL]	12 ± 2	6–16	12 ± 2	11–12	12 ± 2	11–12	0.648
PSA [ng/mL]	470 ± 1114	2–9579	610 ± 1320	295–924	166 ± 154	111–221	0.214
NSE [μg/L]	29 ± 16	13–133	30 ± 17	25–34	29 ± 13	25–34	0.919
GGT [U/L]	62 ± 125	10–931	68 ± 149	32–103	49 ± 34	37–61	0.175
GOT [U/L]	33 ± 33	12–292	36 ± 39	26–45	29 ± 9	25–32	0.514
GPT [U/L]	21 ± 24	5–170	23 ± 28	16–29	19 ± 7	16–21	0.283
**Imaging**							
SUV_max_	69.38 ± 47.30	8.89–276.00	72.65 ± 45.84	61.72–83.58	62.22 ± 50.35	44.06–80.37	0.151
SUV_mean_	8.62 ± 3.44	3.93–27.21	8.95 ± 2.83	8.28–9.63	7.88 ± 4.46	6.27–9.49	**0.003**
TLP [L × SUV]	9.103 ± 9.511	0.128–38.640	9.370 ± 8.841	7.26–11.48	8.519 ± 10.970	4.57–12.47	0.199
MTV [L]	1.101 ± 1.163	0.018–4.963	1.139 ± 1.149	0.86–1.41	1.020 ± 1.209	0.58–1.46	0.387

alkaline phosphatase (ALP); confidence interval (CI); gamma glutamyl transferase (GGT); glutamate oxaloacetate transaminase (GOT); glutamate pyruvate transaminase (GPT); hemoglobin (Hb); molecular tumor volume (MTV); neuron-specific enolase (NSE); prostate-specific antigen (PSA); maximum standardized uptake value of the tumor lesions (SUV_max_); mean standardized uptake value of all tumor lesions (SUV_mean_); total lesion PSMA (TLP).

**Table 3:** Univariat and multivariable Cox regression model for association with OS.

Variable	Univariate Analysis			Multivariable Analysis		
	HR	95% CI	*p*-Value	HR	95% CI	*p*-Value
**Serum**						
ALP	1	1–1.001	0.158			
Hb	0.852	0.75–0.967	**0.013**	0.898	0.778–1.036	0.141
PSA	1	1–1	0.166			
NSE	1.012	0.995–1.03	0.175			
GGT	1	0.998–1.001	0.719			
GOT	1	0.993–1.006	0.98			
GPT	0.991	0.978–1.005	0.22			
**Imaging**						
SUV_max_	0.998	0.992–1.004	0.496			
SUV_mean_	1.001	0.998–1.004	0.416			
TLP [L × SUV]	1.033	1.008–1.059	**0.009**	1.033	1.008–1.059	**0.009**
MTV [L]	1.324	1.088–1.611	**0.005**	1.106	0.622–1.966	0.732

alkaline phosphatase (ALP); confidence interval (CI); gamma glutamyl transferase (GGT); glutamate oxaloacetate transaminase (GOT); glutamate pyruvate transaminase (GPT); hemoglobin (Hb); hazard ratio (HR); molecular tumor volume (MTV); neuron-specific enolase (NSE); prostate-specific antigen (PSA); maximum standardized uptake value of the tumor lesions (SUV_max_); mean standardized uptake value of all tumor lesions (SUV_mean_); total lesion PSMA (TLP).

## Data Availability

The datasets used and analyzed during the present study are available from the corresponding author upon reasonable request.

## Abbreviations

ADT	Androgen deprivation therapy
ARSI	Androgen receptor signaling inhibitor
ALP	Alkaline phosphatase
CI	Confidence interval
CT	Computed tomography
ECOG	Eastern Cooperative Oncology Group
GGT	Gamma glutamyl transferase
GOT	Glutamate oxaloacetate transaminase
GPT	Glutamate pyruvate transaminase
Hb	Hemoglobin
HR	Hazard ratio
m	Months
mCRPC	Metastatic castration-resistant prostate cancer
MIP	Maximum intensity projection
MTV	Metabolic tumor volume
NSE	Neuron specific enolase
PET	Positron emission tomography
PSA	Prostate-specific antigen
PSMA	Prostate-specific membrane antigen
RLT	Radioligand therapy
SD	Standard deviation
SUV	Standardized uptake value
SUV_max_	Maximum standardized uptake value
SUV_mean_	Mean standardized uptake value
TLP	Total lesion PSMA
